# PET/MR Imaging: New Frontier in Alzheimer's Disease and Other Dementias

**DOI:** 10.3389/fnmol.2017.00343

**Published:** 2017-11-01

**Authors:** Xin Y. Zhang, Zhen L. Yang, Guang M. Lu, Gui F. Yang, Long J. Zhang

**Affiliations:** Medical Imaging Center, Jinling Hospital, Medical School of Nanjing University, Nanjing, China

**Keywords:** PET/MR, Alzheimer diseases, clinical applications, technical considerations, PET radiotracers

## Abstract

Alzheimer's disease (AD) is the most common form of dementia; a progressive neurodegenerative disease that currently lacks an effective treatment option. Early and accurate diagnosis, in addition to quick elimination of differential diagnosis, allows us to provide timely treatments that delay the progression of AD. Imaging plays an important role for the early diagnosis of AD. The newly emerging PET/MR imaging strategies integrate the advantages of PET and MR to diagnose and monitor AD. This review introduces the development of PET/MR imaging systems, technical considerations of PET/MR imaging, special considerations of PET/MR in AD, and the system's potential clinical applications and future perspectives in AD.

## Introduction

Alzheimer's disease (AD) is one of the principal health-care challenges experienced globally in today's society. It is reported that nearly 5.4 million Americans have AD (Alzheimer's Association, 2013). It is estimated that 700,000 Americans will die of Alzheimer's disease at an age greater than or equal to 65 years old, and many of which will be due to the complications of AD (Alzheimer's Association, 2016). China is the world's most populous country in aging. After 65 years old, the prevalence of AD will double every 5 years. Furthermore, the prevalence of AD will account for 1/3 of the total population after the age of about 85 years old (Querfurth and Laferla, 2010).

AD is characterized by neurofibrillary entanglement and β-amyloid (Aβ) plaques comprised of tau amyloid fibrils, which is associated with memory impairment and other cognitive problems (Jack et al., 2010; Li et al., 2014). The exact molecular mechanisms behind the associated memory loss in AD is not known. There are reports that some healthy individuals have these plaques and deposits but do not develop memory loss (Pereznievas et al., 2013). There are many known risk factors for AD including the following: age, obesity, diabetes, increasing inflammation in the brain, and infections (Birch et al., 2014). Additional risk factors include some susceptible genes for AD, such as *APOE, CR1, FERMT2*, and *COMT. APOE* genotype is the strongest risk factor for late-onset AD (Karch and Goate, 2015). There are three stages of AD: preclinical AD, mild cognitive impairment (MCI), and dementia, as classified by the National Institute of Aging and the Alzheimer's Disease Association (Dubois et al., 2014). Preclinical AD is defined as personal cognitive impairment and encephalopathy of AD at autopsy. Dementia caused by AD contains three stages ranging from mild to moderate to severe. Preclinical AD is divided into asymptomatic cerebral amyloidosis (Sun et al., 2013), synaptic dysfunction, and amyloid positivity. These three divisions are the proof of neurodegeneration and slight cognitive decline. The evidence of neurodegeneration includes the following: elevated cerebrospinal fluid tau or p-tau; metabolism of the cortex, forebrain, and/or temporal cortex in FDG PET; cortical thinning/gray matter loss in the lateral and medial parietal lobe; posterior cingulate and lateral temporal cortex or the hippocampus atrophy.

The current diagnosis of AD is based mainly on clinical manifestations, disease history, psychological tests, and other adjunct information (Tokuchi et al., 2016). Non-invasive eye tests have been found to help diagnose AD. Moreover, beta-amyloid protein found in the eye is significantly associated with brain beta amyloid protein levels (Bandelow et al., 2011). The refinement of neuroimaging plays a vital role in evaluating patients who are suspected of having AD. MR imaging and PET are two major modalities used to diagnose AD. MR imaging provides structural and functional information of the brain by utilizing detailed regional tissue characterization with a superior soft tissue contrast. This imaging modality can clearly distinguish between cerebral gray matter and white matter, and it can display the brain tissue in a three-dimensional way. PET imaging can provide metabolic and molecular information of the brain. This type of imaging can show metabolic and molecular changes that are not restricted to glucose and Aβ plaque. Considering AD lesions are tangles and plaques, PET has a high sensitivity in mapping the distribution of these lesions. Therefore, these modalities can not only qualitatively diagnose AD, but also quantify the sensitivity of the threshold that can be identified in the normal phase and different stages of AD (Barthel et al., 2015). Thus, combined structural (MRI) and functional (PET) imaging can be used as a better and more accurate diagnostic technique than either of these imaging modalities alone.

In the past couple of decades, hybrid imaging models have been gaining widespread acceptance in clinical practice. These models include PET/computed tomography (PET/CT), fluorescence molecular tomography (FMT)/CT, and single-photon emission computed tomography/CT. The new hybrid model, PET/MR imaging, can make up for the insufficiency of PET/CT in areas such as ionizing radiation, and provide better performance in the AD field. For example, Dixon MR exhibited good soft tissue contrast in attenuation correction and was found to be used for FDG uptake of anatomical localization, especially for the lesions in the soft tissue. Subsequently, PET/MR imaging shows potential in diagnostic classification, disease staging, prognostic evaluation, and understanding of the pathomechanisms of AD (Jadvar and Colletti, 2014). Additionally, obtaining all required imaging and biomarker information within one imaging session, not only provides improved convenience to the patients and their caregivers but also to the referring physicians (Barthel et al., 2015).

This review will introduce the development of PET/MR imaging systems, technical considerations of PET/MR imaging, special considerations of PET/MR imaging in AD, PET/MR technology, clinical applications, and future perspectives in AD.

## The development of PET/MR imaging systems

The development in multimodality imaging devices is a milestone in medical imaging. In the last few decades, PET/MR technology has been developed rapidly. In 1997, Shao et al. successfully acquired simultaneous PET and MR phantom images. They placed lutetium oxyorthosilicate (LSO) crystals in the ring configuration within the receiver coil of the MR imaging system. This prototype PET detector can be paired, based on compatibility, with a clinical MR imaging system and provide both PET and MR images. In 2008, Schlemmer et al. applied MR compatible PET detector technology to acquire simultaneous PET/MR images of the human brain. Following phantom studies, they successfully imaged brain glucose consumption in two patients using ^18^F-FDG-PET, MR imaging, and MR spectroscopy. They demonstrated that PET/MR imaging is feasible in humans, opening new possibilities in new areas of molecular imaging. After decades of development, combining multi-parameter anatomic and functional imaging of MR with molecular imaging of PET is substantial to the scientific and clinical researches of central nervous system, cardiovascular system, and tumors. Table 1 lists the development of several PET/MR imaging systems.

**Table 1:** Development of PET/MR imaging systems.

**Year**	**Authors/companies**	**Features**
1997 (Shao et al., 1997)	Shao et al	First successfully acquired simultaneous PET and MR phantom images and verified the feasibility of simultaneous imaging of PET and MR.
2005 (Pichler et al., 2008)	Siemens	Developed the first human PET/MR scanner and showed its first brain PET/MR image to the North American Radiology Society in 2006.
2008 (Schlemmer et al., 2008)	Schlemmer et al	A PET/MR imaging technique for human skull base. It was proved that PET/MR imaging was feasible in humans, and opened a new possibility for the new field of molecular imaging.
2010 (Delso et al., 2011)	Siemens	The first fully integrated body PET-MRI, Siemens called it Molecular Magnetic Resonance Imaging (Biograph™ mMR). The examination requires only one scan, and the patient did not need to perform MR and PET scans separately, saving time and the cost.
2014 (Seifert et al., 2013)	General Electric	The digital MR compatible silicon photomultiplier detector (SIPM) technology and Turbo time-of-flight (TurboTOF) technology.

## Technical considerations of PET/MR imaging

### Attenuation correction

Attenuation correction is the hinge to quantitative PET reconstruction. MR images do not reflect direct photon attenuation information (Yang and Zhang, 2016). So, finding a reliable alternative to standard transmission attenuation correction for PET data is one of the major technical challenges of integrated PET / MR imaging. Dickson et al. clearly reinforced these issues for neurological imaging that attenuation correction limits the quantitative accuracy of neurological PET/MR imaging (Dickson et al., 2014). In order to overcome these challenges, many efforts have been made to perform attenuation correction in PET/MR imaging systems, such as segmented 2-point Dixon MR imaging sequence, ultrashort echo time (UTE) sequences, atlas-based approach, machine learning approach, PET/MR imaging systems with time-of-flight (TOF) technology, and the use of non-MR information (Visvikis et al., 2014). Table 2 lists some attenuation correction methods of PET/MR imaging systems with their advantages and disadvantages.

**Table 2:** Attenuation correction methods of PET/MR imaging.

**Approach**	**Advantages**	**Disadvantages**
Segmented 2-point Dixon MR imaging sequence (Werner et al., 2015)	Standard method in processing PET data obtained from fully integrated PET/MR imaging. Reduces the undervalue bony tissue from −25.5 ± 7.9% (Dixon) to −4.9 ± 6.7% to enhance the PET quantification in PET/MR imaging of the whole body, particularly in tissue close to bone.	Ignores the specific contribution of bone to photon attenuation. It is reported that the resulting PET signals range from 11 to 25% (Navalpakkam et al., 2013)
Ultrashort echo time (UTE) sequence (Cabello et al., 2015)	Significantly reduced errors in quantification of radiotracer takeup; delivering a more accurate PET image quantification for an improved diagnostic workup in dementia patients.	Susceptible to misclassification.
PET/MR imaging systems with TOF technology (Rezaei et al., 2014)	TOF data can be used for transmitting data, as well as for attenuating graphs.	The algorithm must maintain the tracer distribution and the attenuation factors in memory during reconstruction of the project image.
Atlas-based and machine learning approaches (Arabi and Zaidi, 2016)	Extra error decrease of the order of less than 5%. Sorted atlas pseudo-CT (SAP) to estimate bone and lung attenuation accurately.	Checking in errors and dissection discrepancies among patients.
Non-MR information for PET AC in PET/MR imaging (Defrise et al., 2014)	The most potential solution for future TOF-based PET/MR scanners.	The limited availability of a combined PET/MR scanners presents some of the relevant engineering challenges of clinical implementation, which is more suitable only for sequential PET/MR systems.

### Head motion correction

It is hard to prevent head motion in patients with neurologic disorders such as AD. A large amount of head motion can cause debasement of PET images and critical artifacts. There are some methods to restrain the subject's head motion when only using PET scanners (Henriksen et al., 2016). There is a simple technique which can achieve a frame-by-frame correction by adjusting the reference positions of individual frames and summarizing the quantities they create. Additionally, the Polaris optical tracking system can also be used to correct for head motion. This system is combined with the line-of-response rebinning algorithm.

In combined PET/MR imaging scanners, simultaneous PET data and MR imaging data may possibly gain high time resolution motion estimation, eliminating the necessity of optical tracking systems. Catana et al. (2011) initially used a Hoffman phantom to study the precision of the motion correction algorithm. Manber et al. also showed that head motion associated with a fixed multi-channel coil had adverse effects on prospective motion correction of functional MR imaging, and adjusting the susceptible map at 3.0T would slow this effect (Faraji-Dana et al., 2016). Then, Lin et al. proposed a motion correction technique in multicoil imaging applications, including data collection and reconstruction. It was a bit-reversed radial acquisition scheme, with a combination of fast self-calibration of parallel imaging method, automatic calibration part of the generalized parallel acquisition (GRAPPA), and operator for wider radial bands (GROWL). The method did not prolong the scanning time and was suitable for short, repeated time series (Lin et al., 2012).

MR imaging-based motion correction algorithm could also be implemented in an integrated PET/MR imaging scanner for PET motion correction to improve the quality of PET image and benefit many nervous system applications. Catana et al. proposed a new algorithm for MR imaging, compatible with data processing and rigid-body motion correction. They found the motion induced by high temporal resolution MR imaging can be applied to PET motion correction. Therefore, it is possible to improve PET image quality, increase their dependability, repeatability, and quantitative accuracy based on MR imaging-based motion correction algorithm (Catana et al., 2011).

## Special considerations of PET/MR imaging in AD

Corrections for brain atrophy and partial-volume effects are special considerations of PET/MR imaging in AD when evaluating the condition with ^18^F FDG PET. It is requisite to exploit and carry out effective correction of brain atrophy and partial-volume effects which might bring out false positive of the PET diagnosis. The “hot spot” imaging of amyloid associated with brain atrophy in AD is contrary to the PET signal and potentially reduces the PET diagnostic ability. Yanase et al. (2005) used a Hoffman 3D brain phantom to verify the feasibility of a newly developed partial-volume effect correction method. The experiment was performed on 139 healthy, Japanese volunteers and PET images were corrected for partial-volume effects with gray matter volume. They found the PET image provided more homogeneous tracer distribution in the whole phantom with correction method than that without correction method. Thus, such a method can improve PET image quality and increase its reliability. Rullmann et al. verified that quantitative ^18^F-florbetaben PET scans can correct the partial volume effect, especially in assessing patients with brain atrophy (Rullmann et al., 2016).

## PET/MR technology in AD

PET/MR has been concluded as “Anatomy + Activity + Connectivity” in the Neurology field. The combination of PET and MR shows an extremely attractive prospect.

### PET radiotracers in AD

The main pathology of AD includes Aβ deposition, tau pathology or neurofibrillary tangles, and degeneration or damage of AD like neurons. The PET tracer most often employed in routine clinical practice is ^18^F-FDG. It is transported into intracellular space by glucose 1 transporters, followed by hexokinase phosphorylation. Typical AD is associated with a characteristic topographic pattern of FDG-PET hypometabolism that appears earliest and most severely in the medial parietal and lateral temporal-parietal cortex. Aβ deposition is the most concerning pathologic aspect of AD, while ^11^C-PiB is the most commonly used *in vivo* amyloid PET tracer (Bhogal et al., 2013). It has been demonstrated that toxicity of Aβ is tau dependent; thus, targeting tau is an important strategy to *in vivo* image AD. Some radiotracers targeting tau, such as ^18^F-THK5351, ^18^F-FDDNP, ^18^F-MK-6240, ^18^F-T807, ^18^F-AV-1451, have been successfully developed. In addition, other radiotracers such as ^18^F-SAHA, ^64^Cu-CUDC, ^18^F-florbetapir, ^18^F-florb tetaben, and ^18^F-flutemetamol (Rocchi et al., 2015) are used in AD cases. Table 3 lists some useful radiotracers of PET for AD.

**Table 3:** PET radiotracers in AD.

**Targeting**	**Radiotracers**	**Features**
Amyloid-β	^18^F-florbetapir, ^18^F-florbetaben, ^18^F-flutemetamol	Shows or excludes brain amyloid load in MCI, a prodromal phase of dementia syndromes, and early-onset clinical presentation of AD-like dementia (Johnson et al., 2013)
	^18^F-AZD4694 (Zsolt et al., 2012)	An evaluation of therapeutic purposes and disease modifying therapies. The use of radioisotope ^18^F as a marker ligand has broad clinical potential. ^18^F-azd4694 meets the requirements for the diagnosis of amyloid ligands and the evaluation of AD disease modifying therapies.
	^18^F-BAY94-9172 (Rowe et al., 2008)	Abeta images should be early diagnosed, but the short half-life of the current Abeta specific ligand is a hindrance for clinical use. ^18^F-bay94-9172 is a Abeta ligand for clinical use because of its long half-life of 18F.
	^18^F-RAGER (Cary et al., 2016)	The advanced glycation end products receptor (RAGE) is believed to mediate cyclic amyloid beta entry into the brain and amplify beta induced disease. ^18^F-rager microPET angiography confirmed CNS penetration and increased uptake of regions known to express anger in the brain.
	^18^F-NAV4694 (Zimmer et al., 2013)	It is a amyloid imaging agent with a higher binding of ^18^F-NAV4694 in post-mortem AD brains
	^11^C-PiB (Rinne et al., 2010)	Tracks amyloid plaques *in vivo*. ^11^C-PiB PET appears to be useful in assessing the impact of amyloid beta loading on cortical fibers in the treatment of underlying Alzheimer's disease.
	^11^C-TAZA (Pan et al., 2016)	TAZA affinity to 5 times stronger than PIB. ^11^C-TAZA is related to plaques in the brain of AD, and the ratio of gray matter to white matter is greater than 20. ^11^C- TAZA is highly integrated with human AD hippocampal plaques.
	^11^C-SB-13 PET (Verhoeff et al., 2004)	^11^C-SB-13 is an effective PET tracer with similar properties with ^11^C-PiB for fibrillar Abeta imaging *in vivo*.
	^11^C-AZD2184 (Nyberg et al., 2009)	Low non-specific binding, reversible binding, and high signal-to-noise ratio were apparent in early peak equilibrium.
Glucose metabolism	^18^F-FDG (Mosconi et al., 2008)	The best method for studying the characteristics of brain metabolic imaging in AD, May provide an objective and sensitive support to the clinical diagnosis in early dementia. It can differential diagnosis of the major neurodegenerative disorders, including mild and moderate-to-severe dementia patients and MCI.
Tau protein	^18^F-THK5351 (Harada et al., 2015)	High affinity of hippocampal homogenates in AD brains and rapid separation from white matter tissue. THK5351 binds to neurofibrillary tangles with a high selective and high signal-to-background ratio. It is a useful PET tracer for early detection of nerve fiber lesions in patients with AD.
	^18^F-FDDNP (Buongiorno et al., 2017)	Marking senile plaques and neuronal fibrous entanglement specificity. Being as a dementia risk biomarker.
	^18^F-MK-6240 (Collier et al., 2017)	It is a potent and selective tau tracer with great binding potential which can detect human neurofibrillary tangles.
	^18^F-T807, ^18^F-AV-1451 (Shcherbinin et al., 2016)	^18^F-T807 is a PET radiotracer developed for imaging tau protein aggregates, which is implicated in neurological disorders including AD. The early separation of cortical and cerebellar temporal activity curves, as well as slow and spatially inhomogeneous gaps from the cortical region, can be observed.
Microglial activation	^11^C-PK11195 (Schuitemaker et al., 2012)	The selective ligand transport protein (18 kDa) (TSPO) is highly conveyed by activated macrophages. TSPO expression is upregulated in activated microglial cells in response to inflammation or injury to the brain. It may compose a biomarker of brain inflammation and reactive gliosis. Increasing the binding of TSPO ligands reflects increased microglial activation is a critical event in inflammatory response. Imaging of chronic inflammation is associated with an ideal TPSO tracer for its anti-inflammatory response.
	^18^F-DPA-714	A marker of microglial activation which is a valuable innovative tool for the accurate evaluation of early and preclinical AD (Hamelin et al., 2016). The protective effect of early microglial cell reaction on AD is of clinical significance in promoting targeted therapy of microglia (Shechter and Schwartz, 2013).
	^18^F-FEMPA (Varrone et al., 2015).	18 kDa TSPO is a potential tool for studying microglial activation and inflammation in early AD. ^18^F-fempa is a new type of high affinity ligand, two generation TSPO, with suitable pharmacokinetic properties. ^18^F-fempa seems to increase the detection of TSPO binding in patients with AD with suitable ligands.
Hydroxamic acid	^18^F-FAHA, ^18^F-SAHA (Tang et al., 2014)	They showed a great potential for assessing the (Histone deacetylase) HDAC activity of brain in AD. They can also be applied in hematologic malignancy and solid tumors.
P-glycoprotein	^18^F-MC225 (Aggarwal et al., 2015)	P-glycoprotein is a protective efflux transporter of the blood-brain barrier, which exhibits altered functions in many neurological diseases. ^18^F-MC225 is a useful radiotracer in blood brain barrier measures, especially P- glycoprotein function.
High tissue transglutaminase (TG2) crosslinking activity	C11 labeled acryl amides (Van der Wildt et al., 2016)	High tissue transglutaminase (TG2) crosslinking activity has been implicated in the pathogenesis of various diseases. The specific development of an PET tracer for active TG2 is further explored *in vivo* and provides a new tool for TG2 biology in disease states.

### MR imaging techniques in AD

Advanced MR imaging techniques have been used more and more in neuropsychiatric diseases during the past few decades. A series of MR imaging sequences can be developed to support PET interpretation and to exclude or identify clinically relevant pathologic conditions, e.g., excluding structural lesions that may be treated neurosurgically, and excluding generalized or local edema, etc. (Visvikis et al., 2014). Advanced functional MR imaging sequences, such as diffusion weighted imaging (DWI), diffusion tensor imaging (DTI), perfusion weighted imaging, blood oxygen level dependent functional MR imaging (BOLD-fMRI) (Galvin et al., 2011) and MR spectroscopy (MRS), (Falini et al., 2005) can be routinely used in PET/MR imaging system. Sala-Llonch et al. conducted an integrated multi-modal MRI study using task functional MR imaging, high resolution structural MR imaging and DTI. This study confirmed the importance of early integrated multimodal MR imaging studies. In their study, they identified early functional abnormalities in critical brain areas of the default mode network and the precise anatomical substrates, an early neuroimaging marker that may reflect AD (Sala-Llonch et al., 2011). Table 4 lists these common MR imaging sequences with their advantages for AD applications.

**Table 4:** MR sequences used in AD studies.

**Sequences**	**Features**
Three-dimensional (3D) T1-weighted magnetization-prepared rapid acquisition gradient-echo(T1-MPRAGE) sequence	Particularly well suited for evaluation of structural pathology and regional brain atrophy, for example, the hippocampal structures.
T2-weighted fluid attenuation inversion recovery (FLAIR) sequence and T2-weighted BLADE or turbo spin-echo (TSE) sequence.	Sensitive for detection of edema, demyelination, and ischemic changes; and are important in identifying neoplasms and cerebrovascular disease.
Susceptibility-weighted imaging (SWI) sequence or gradient-echo T2^*^-weighted sequence	Sensitive to blood products and are useful to identify cerebral microbleed.
Diffusion weighted imaging and diffusion tensor imaging (DTI)	Measuring diffusion properties of water molecules. Identify brain microstructure changes which is hard to find in traditional CT and MR imaging, especially the nerve fiber bundle changes and direction.
Diffusion kurtosis imaging	Neuronal fibers at the intersection can be shown better. Diffusion kurtosis is helpful to improve the microstructure (Hui et al., 2012), distinguish AD from MCI (Benitez et al., 2013)
Perfusion imaging	Traditionally using contrast-enhanced MR perfusion or ASL without contrast media administration, which is another attractive approach especially for combined brain PET/MR imaging (Schaefer et al., 2014). Dynamic contrast-enhanced MRI can provide the tissue perfusion information.
Magnetic transfer imaging (MTI) (Abdel-Fahim et al., 2014)	Specific pathological information of brain injury was increased and small abnormalities were detected in normal brain tissues.
MR spectroscopy (MRS) (Falini et al., 2005)	MRS is one of the key applications in high field MR systems. The MRS will be obtained from improving the signal-to-noise ratio and enhancing the spectral resolution.
Blood oxygen level dependent functional MR imaging (BOLD-fMRI).	Includes task- and resting-state functional MR imaging, which can provide neurobiological basis underlying brain structures and functions, serve an early marker for the diagnosis of AD and evaluate AD treatment efficacy (Galvin et al., 2011)
3D TOF MRA (Bogunović et al., 2011)	It can clearly show the intracranial arterial vessels without administration of contrast media.

3-T high-field MRI has been used in AD studies in clinical practice. In recent years, there have been AD studies reported using 7-T MRI scanners. Martin et al. have demonstrated that simultaneous PET and MR imaging in a 7 Tesla system is feasible (Judenhofer et al., 2007). Compared with 3-T MRI, 7-T MRI has some advantages including increased signal noise ratio and the enhanced sensitivity to susceptibility. 7-T MRI can accurately detect iron deposits within activated microglia, which may help shed light on the role of the immune system in AD pathogenesis (Ali et al., 2015). 7-T MRI overcomes the limitations of suboptimal resolution, having a potential to precisely detect neuroanatomical atrophy in imaging neuroanatomy of AD. Furthermore, 7-T MRI may also play a promising role in the detection of microinfarcts, which may help further elucidate the relationship between cerebrovascular health and AD progression. However, MRI susceptibility effects increase linearly with field strengths, which causes increased susceptibility artifacts and signal dropouts due to off-resonance frequencies (Krug et al., 2007). Additionally, 7-T MRI has not been widely used in routine clinical practice.

### PET/MR imaging workflow in AD

At present, PET/MR imaging is a hot spot of research in AD. The accuracy of imaging results comes from the combination of information from both PET and MR. In typical brain PET/MR imaging protocols, MR imaging may take up to 60 min while a PET scan may take 15 min. There are at least a few minutes between the tracer injection and the interval between the MR contrast agents to prevent interaction. Thus, MR examination time usually determines the total time of PET/MR imaging study. In addition, the injection of MR contrast agents is tightly coupled with a single MR sequence in the mixed imaging protocols (von Schulthess and Veit-Haibach, 2014) Figure 1 shows an example of PET/MR imaging workflow (Werner et al., 2015). Bailey et al. pointed out that effectively optimizing the patient preparation and acquisition process is a major challenge. Single-injection single time point imaging using PET/MR imaging only is an effective optimization (Bailey et al., 2014).

**Figure 1:**
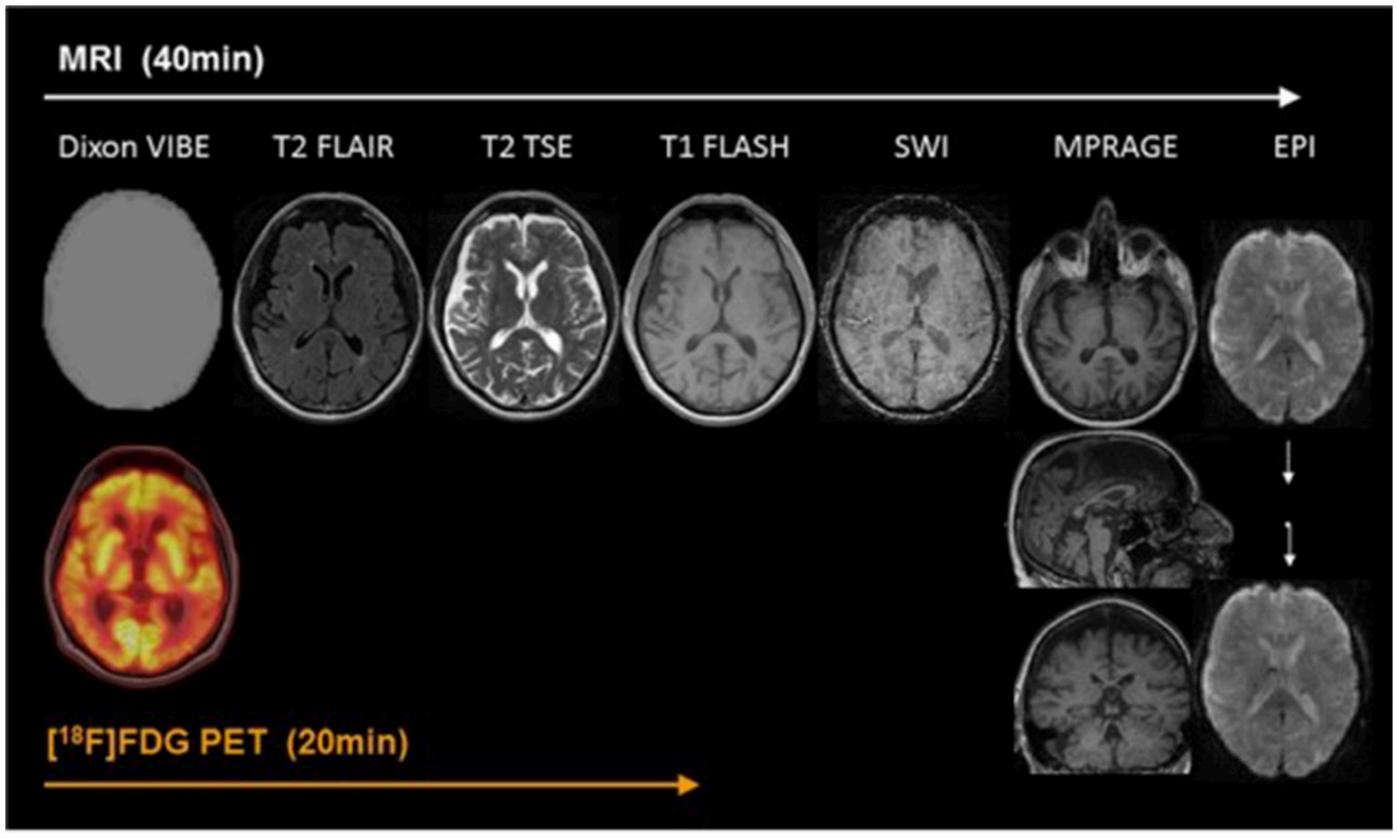
Example of simultaneous PET/MRI workflow in dementia. The workflow of PET/MR includes scanning of MR and PET. In the diagram, Dixon VIBE for PET data attenuation correction and other six sequences are shown in turn. MR scans take 40 min, PET takes only 20 min, and MR scans take up most of the process. With permission, from reference 33.

## Clinical applications of PET/MR imaging in AD

### PET/MR imaging for early diagnosis of AD

**Figure 2:**
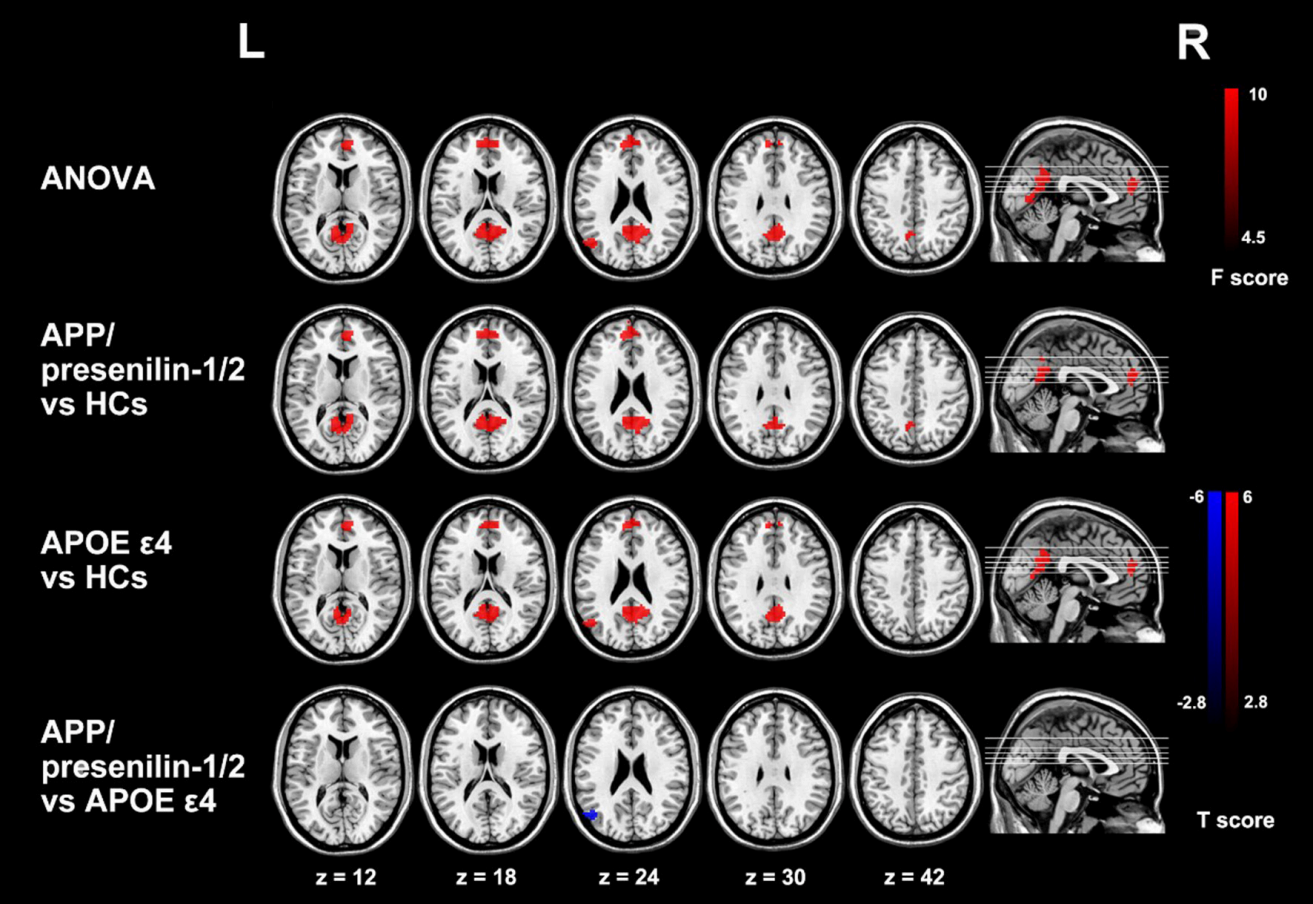
Group differences of functional connectivity based on the seed of right hippocampus in resting state fMRI. The three groups have differences in the medial prefrontal cortex (mPFC), the anterior lobe and the left posterior gyrus (MTG). Comparing with the control group, the functional connectivity of the medial prefrontal cortex and the anterior lobe is enhanced with the right hippocampus in APP/presenilin-1/2 and APOE ε4 carriers. With permission, from reference 39.

There are two effective imaging markers in AD: hippocampal atrophy and cerebrospinal fluid (CSF) biomarkers (Frisoni et al., 2010). ^18^F-FDG PET is another diagnostic marker in the revised diagnostic criteria of AD. The temporal region is also an effective marker of neurodegenerative diseases associated with synaptic dysfunction and can be used as a marker for early diagnosis of AD (Sander et al., 2013). The most striking feature of clinically diagnosed AD in structural MR imaging is volume loss of medial temporal lobe, evident in the hippocampus and entorhinal cortex (Appel et al., 2009). Hippocampus volume loss, which discriminates AD from healthy controls with high accuracy, is considered the best established neuroimaging biomarker of AD (Apostolova et al., 2007). Functional MR imaging also has a tremendous potential to provide the early diagnosis of AD (Figure 2; Zheng et al., 2017).

Goubran et al. used ^18^F-FDG PET/MR imaging to assess FDG activity and arterial spin-labeling (ASL) MR to assess cerebral blood flow (CBF) in patients with MCI and AD. They found FDG PET metabolism was significantly reduced in AD and MCI patients (Goubran et al., 2015). Fan et al. used both ASL MRI and 15O water PET to achieve a better diagnosis of MCI (Fan et al., 2007). Binnewijzend et al. investigated ASL-derived CBF changes in pre-dementia stages of AD and concluded that the continuing decline of CBF along the continuum of AD suggested the potential application of ASL-CBF as a measure for disease progression (Binnewijzend et al., 2016).

Decreased amyloid-β1-42 (Aβ42) in CSF and increased Aβ tracers in the brain on PET are considered to be the earliest biomarkers of AD. Lewczuk et al. assayed Aβ42 and Aβ40 using two recently developed immunoassays and they found that as a marker of amyloid positive, CSF Aβ42/40 was better than Aβ42 alone by PET (Lewczuk et al., 2017). The default mode network activity associated with AD-specific pathology may also be an early feature of AD. Celebi et al. studied cognitive profiles, default mode network connectivity alterations, CSF amyloid beta (Aβ) 1–42, total tau, phosphorylated tau 181, and a-synuclein levels. They showed that default mode network activity changes in AD were closely related to cognitive functions and Aβ pathology (Celebi et al., 2015). Recently, Zhang et al. proposed an eigenbrain to detect AD brains. They extended the eigenbrain to 3D and proved the effectiveness of the proposed eigenbrain to 3D by detecting objects and brain regions related to AD (Zhang et al., 2016).

### PET/MR imaging for differential diagnosis of AD

**Figure 3:**
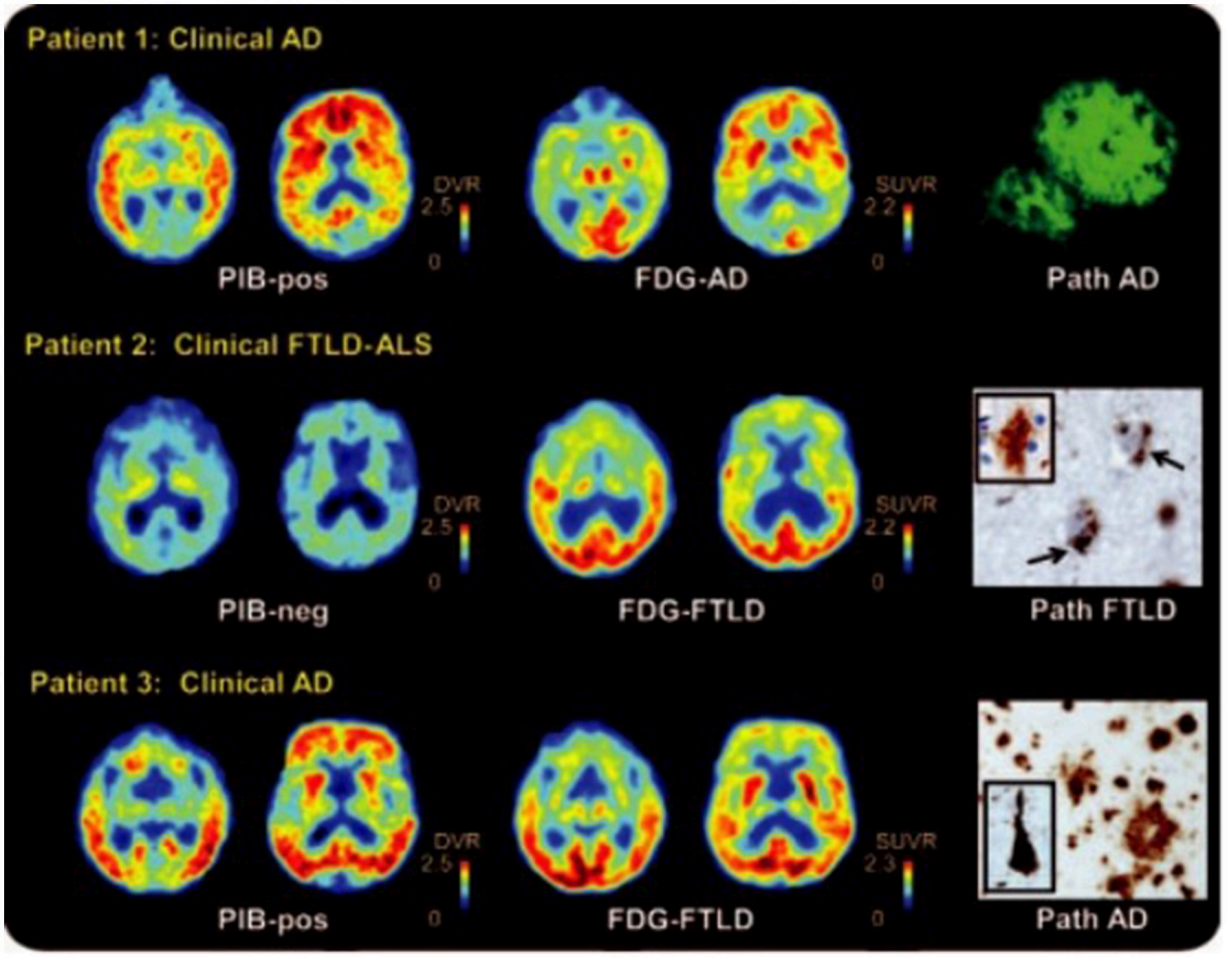
^11^C Pittsburgh compound B (PiB), 18F-fluorodeoxyglucose (FDG) PET, and histopathology in selected patients. Patient 1 had clinical AD with diffuse cortical and striatal PIB, and predominantly temporal regions of the FDG. Post-mortem examination showed amyloid plaques in the temporal cortex. Patient 2 had frontotemporal lobar degeneration [FTLD]–amyotrophic lateral sclerosis [ALS]. The major metabolic loss in frontal lobe is manifested in this patient. Pathological analysis reveals diffuse plaques of early Aβ pathology. Patient 3 had clinical AD and was positive for PiB, but FDG showed decreased frontal lobe metabolism. Autopsy demonstrated AD diagnosis with frequent neuritis and neurofibrillary pathology. With permission, from reference 47.

FDG PET can be used to differentiate AD from frontotemporal lobar degeneration. Reiman et al. (2011) studied the patients with AD and frontotemporal dementia who receive PiB and FDG-PET and concluded that PiB and FDG showed the similar precision in the differential diagnosis of AD and frontotemporal lobar degeneration, while PiB slightly outperformed FDG in detecting AD patients (Figure 3; Rabinovici et al., 2011).

PET/MR imaging can provide the differential diagnosis information of AD by providing neuroimaging biomarkers of amyloid pathology and neuronal injury. Barthel et al. employed a typical simultaneous brain PET/MR imaging acquisition protocol to image dementia and made the differential diagnosis of AD, dementia with Lewy bodies (DLB), and vascular dementia (VaD) (Barthel et al., 2016). PET/MR can provide very useful diagnostic information for AD, frontotemporal dementia,semantic dementia, and patients with posterior cortical atrophy. To detect or exclude nonneurodegenerative disease, MR imaging is a major prerequisite for the differential diagnosis of various dementias (Drzezga et al., 2014). The study conducted by Henriksen et al. (2016) was composed of 4 standard MR imaging sequences and a simultaneous 10-min PET acquisition which was performed 40 min after injection of 200 MBq FDG. The patient was initially diagnosed with semantic dementia, a variant of frontotemporal lobar degeneration, but atypical AD was considered as a differential diagnosis. In the imaging, MR imaging identified a lacunar infarct in the right internal capsule and a single microbleed in the right thalamus. Both abnormalities had minor or no effects on the regional metabolic activity seen in ^18^F-FDG PET. The hippocampal volumetric report showed marked bilateral reduction in the total hippocampal volume and a marked increased inferior lateral ventricular volume with significant asymmetry, where the left hippocampal volume was only 70% of that on the right side. ^18^F-FDG PET showed the same asymmetry in the anterior temporal lobes, along with moderate to severe metabolic reduction extending posteriorly to involve the temporal and parietal lobes, including the left posterior cingulate cortex. A supplementary amyloid PiB PET scan was negative, indicating semantic dementia rather than atypical AD (Tahmasian et al., 2015b).

### PET/MR imaging for therapy monitoring of AD

Combined structural and functional MR imaging and PET can permit the partial volume effect correction and evaluate the amount of PET tracer taken up a given brain volume. Furthermore, for the various imaging biomarkers observed during different time periods, multimodal assessment can allow much better-informed quantification of disease progress or therapy effect at several scales (Drzezga et al., 2014). Fei et al. used combined MR and PET images to study the method of image registration to improve tumor surveillance. It was concluded that the registration of high resolution MR and PET images of RIF-1 tumor mice may be useful in binding anatomical and functional information, in addition to having potential applications in photodynamic therapy (Fei et al., 2004). Cline et al. investigated the efficacy of nonamyloid targeting microtubule stabilizers paclitaxel in imaging and cognitive testing in transgenic mice with the combination of PET and MR imaging. They concluded that cognitive and imaging tests can distinguish disease from controls and detect outcome improvements from drug therapy (Cline et al., 2015).

## Future perspectives

Although PET/MR imaging has a great increase in the last years, many research and application fields of AD are in need of urgent development in future. PET/MR imaging should be used to investigate the relationship between molecular and metabolic information provided by PET and the anatomy, function, and blood flow information of multimodal MR imaging - which is the most important aspect of PET/MR imaging in future AD studies.

Exploring the synergistic potential of the new PET tracers described together with new MR techniques can answer the question of whether simultaneous PET and MR imaging data acquisition provides synergies over separate data acquisition in dementia imaging. Hybrid PET/MR imaging is useful for assessing brain function, structural function, metabolism, and molecular information at the same time. In the past few decades, there has be question whether or not direct comparison between 15O water PET and ASL MR perfusion imaging for measuring CBF was possible. Hybrid PET/MR imaging made such a comparison feasible. Zhang et al. used a 3T PET/MR imaging with the simultaneous acquisition of pseudo-continuous ASL MR perfusion imaging and 15O water PET in 10 young healthy males. They found simultaneous measurements of CBF were feasible using 15O water PET and ASL-MRI (Zhang et al., 2014). Tahmasian et al. studied 21 patients with MCI and 26 healthy controls who performed simultaneous integrated PET / MR scan. They found that the lower connectivity between the hippocampus and the anterior lobe of the AD dementia patients, the higher the rate of hippocampal metabolism. They concluded that decreased intrinsic connectivity between the hippocampus and the anterior lobe of the hippocampus is associated with higher metabolism of the hippocampus in AD patients (Tahmasian et al., 2015a).

Improving multimodal data analysis is of major interest for combined PET/MR imaging in patients with AD in the big data era. Combining MR imaging with PET imaging information might greatly improve the accuracy of AD diagnosis. Machine learning methods have been introduced into the field of AD, improving the diagnostic accuracy of AD compared to conventional analysis methods. Dukart et al. (2013) systematically applied the whole brain and support vector machines based on regions of interest; improving the detection and discrimination ability of different types of dementia by using the separation and combination of information in different imaging modalities. Their studies show that PET/MR imaging information has greatly improved diagnostic accuracy and suggest that this approach be incorporated into clinical diagnosis and differential diagnostic procedures for neurodegenerative diseases. Dukart et al. (2011) validated that the application of support vector machine classification based on combined information from MRI and ^18^F FDG PET improved the detection and differentiation of AD and frontotemporal lobar degeneration. These results suggest that support vector machine classification based on quantitative meta-analysis of multicenter data is an effective way to diagnose AD. Besides, a major simplification of gaining correct biomarker information in dementia diseases is expected by employing this technique in a one-stop-shop fashion. This technique, through combining amyloid PET/MR imaging, can demonstrate new useful biomarker of AD in the future.

## Conclusions

In summary, PET/MR imaging is a great technological invention. It has some unique advantages to improve early and differential diagnosis of AD through combining the metabolic and molecular information from PET and structural, functional information from MR imaging. With new emerging PET radiotracers and MR imaging techniques, PET/MR imaging has a promising potential to broaden the diagnostic power and become a conventional first-line one-stop-shop clinical imaging tool to improve our understanding of AD.

## Author contributions

XZ and ZY for study design, literature search, and manuscript editing. GL, GY, and LZ for study design, literature search and manuscript revision.

### Conflict of interest statement

The authors declare that the research was conducted in the absence of any commercial or financial relationships that could be construed as a potential conflict of interest.

## References

[B1] Abdel-Fahim R. Mistry N. Mougin O. Blazejewska A. Pitiot A. Retkute R.. (2014). Improved detection of focal cortical lesions using 7T magnetisation transfer imaging in patients with multiple sclerosis. Mult. Scler. Relat. Disord. 3, 258–265. 10.1016/j.msard.2013.10.004 25878014

[B2] Aggarwal N. T. Shah R. C. Bennett D. A. (2015). Alzheimer's disease: unique markers for diagnosis & new treatment modalities. Indian J. Med. Res. 142, 369–382. 10.4103/0971-5916.169193 26609028 PMC4683821

[B3] Ali R. Goubran M. Choudhri O. Zeineh M. M. (2015). Seven-Tesla, MRI and neuroimaging biomarkers for Alzheimer's disease. Neurosurg. Focus 39, E4. 10.3171/2015.9.FOCUS15326 26646928

[B4] Alzheimer's Association (2013). 2013 Alzheimer's disease facts and figures. Alzheimers Dement. 9, 208–245. 10.1016/j.jalz.2013.02.003 23507120

[B5] Alzheimer's Association (2016). 2016 Alzheimer's disease facts and figures. Alzheimers Dement. 12, 459–509. 10.1016/j.jalz.2016.03.001 27570871

[B6] Apostolova L. G. Steiner C. A. Akopyan G. G. Dutton R. A. Hayashi K. M. Toga A. W.. (2007). Three-dimensional gray matter atrophy mapping in mild cognitive impairment and mild Alzheimer disease. Arch. Neurol. 64, 1489–1495. 10.1001/archneur.64.10.1489 17923632 PMC3197839

[B7] Appel J. Potter E. Shen Q. Pantol G. Greig M. T. Loewenstein D.. (2009). A comparative analysis of structural brain mri in the diagnosis of Alzheimer's disease. Behav. Neurol. 21, 13–19. 10.1155/2009/103123 19847041 PMC5444287

[B8] Arabi H. Zaidi H. (2016). Magnetic resonance imaging-guided attenuation correction in whole-body PET/MRI using a sorted atlas approach. Med. Image Anal. 31, 1–15. 10.1016/j.media.2016.02.002 26948109

[B9] Bailey D. L. Barthel H. Beuthin-Baumann B. Beyer T. Bisdas S. Boellaard R.. (2014). Combined PET/MR: where are we now? Summary report of the second international workshop on PET/MR imaging April 8-12, 2013, Tubingen, Germany. Mol. Imaging Biol. 16, 295–310. 10.1007/s11307-014-0725-4 24668195

[B10] Bandelow S. Clifford A. Wardt V. V. D. Gale A. (2011). Accurate non-invasive diagnoses of Alzheimer's disease using eye scanning. Alzheimers Dement 7, S155–S156. 10.1016/j.jalz.2011.05.419

[B11] Barthel H. Schroeter M. L. Hoffmann K. T. Sabri O. (2015). PET/MR in dementia and other neurodegenerative diseases. Semin. Nucl. Med. 45, 224–233. 10.1053/j.semnuclmed.2014.12.003 25841277

[B12] Barthel H. Werner P. Rullmann M. Mildner T. Tiepolt S. Gertz H. (2016). ASL as a substitute for [18F] FDG? A simultaneous brain PET/MRI study. J. Nucl. Med. 57(Suppl. 2), 235.

[B13] Benitez A. Fieremans E. Jensen J. H. Falangola M. F. Tabesh A. Ferris S. H.. (2013). White matter tract integrity metrics reflect the vulnerability of late-myelinating tracts in Alzheimer's disease. Neuroimage Clin. 4, 64–71. 10.1016/j.nicl.2013.11.001 24319654 PMC3853114

[B14] Bhogal P. Mahoney C. Graeme-Baker S. Roy A. Shah S. Fraioli F.. (2013). The common dementias: a pictorial review. Eur. Radiol. 23, 3405–3417. 10.1007/s00330-013-3005-9 24081643

[B15] Binnewijzend M. A. A. Benedictus M. R. Kuijer J. P. A. Flier W. M. V. D. Teunissen C. E. Prins N. D.. (2016). Cerebral perfusion in the predementia stages of Alzheimer's disease. Eur. Radiol. 26, 506–514. 10.1007/s00330-015-3834-9 26040647 PMC4712243

[B16] Birch A. M. Katsouri L. Sastre M. (2014). Modulation of inflammation in transgenic models of Alzheimer's disease. J. Neuroinflammation. 11, 25. 10.1186/1742-2094-11-25 24490742 PMC3922595

[B17] Bogunović H. Pozo J. M. Villauriol M. C. Majoie C. B. van den Berg R. Gratama van Andel H. A.. (2011). Automated segmentation of cerebral vasculature with aneurysms in 3DRA and TOF-MRA using geodesic active regions: an evaluation study. Med. Phys. 38, 210–222. 10.1118/1.3515749 21361189

[B18] Buongiorno M. Antonelli F. Compta Y. Fernandez Y. Pavia J. Lomena F. (2017). Cross-sectional and longitudinal cognitive correlates of FDDNP PET and CSF Amyloid-β and Tau in Parkinson's Disease. J. Alzheimers Dis. 55, 1261–1272. 10.3233/JAD-160698 27814297

[B19] Cabello J. Lukas M. Förster S. Pyka T. Nekolla S. G. Ziegler S. I.. (2015). MR-based attenuation correction using ultrashort-echo-time pulse sequences in dementia patients. J. Nucl. Med. 56, 423–429. 10.2967/jnumed.114.146308 25678486

[B20] Cary B. P. Brooks A. F. Fawaz M. V. Drake L. R. Desmond T. J. Sherman P. (2016). Synthesis and evaluation of [(18)F]RAGER: a first generation small-molecule PET radioligand targeting the receptor for advanced glycation endproducts. ACS Chem. Neurosci. 7, 391–398. 10.1021/acschemneuro.5b00319 26771209 PMC5682588

[B21] Catana C. Benner T. Van der Kouwe A. Byars L. Hamm M. Chonde D. B.. (2011). MRI-assisted pet motion correction for neurologic studies in an integrated MR-PET scanner. J. Nucl. Med. 52, 154–161. 10.2967/jnumed.110.079343 21189415 PMC3125596

[B22] Celebi O. Uzdogan A. Oguz K. K. Has A. C. Dolgun A. Cakmakli G. Y.. (2015). Default mode network connectivity is linked to cognitive functioning and CSF Aβ1-42 levels in Alzheimer's disease. Arch. Gerontol. Geriatr. 62, 125–132. 10.1016/j.archger.2015.09.010 26515126

[B23] Cline M. Cross C. Murra D. Yumul J. Garwin G. Minoshima S. (2015). Use of FDG-PET and MR imaging with cognitive testing in AD therapeutic development. J. Nucl. Med. 56(Suppl. 3), 416.

[B24] Collier T. L. Yokell D. L. Livni E. Rice P. A. Celen S. Serdons K. (2017). cGMP production of the rdiopharmaceutical [18F]MK-6240 for PET imaging of human neurofibrillary tangles. J. Labelled Comp. Radiopharm. 60, 263–269. 10.1002/jlcr.3496 28185305

[B25] Defrise M. Rezaei A. Nuyts J. (2014). Transmission-less attenuation correction in time-of-flight PET: analysis of a discrete iterative algorithm. Phys. Med. Biol. 59, 1073–1095. 10.1088/0031-9155/59/4/1073 24504259

[B26] Delso G. Fürst S. Jakoby B. Ladebeck R. Ganter C. Nekolla S. G.. (2011). Performance measurements of the Siemens mMR integrated whole-body PET/MR scanner. J. Nucl. Med. 52, 1914–1922. 10.2967/jnumed.111.092726 22080447

[B27] Dickson J. C. O'Meara C. Barnes A. (2014). A comparison of CT- and MR-based attenuation correction in neurological pet. Eur. J. Med. Mol. Imaging. 41, 1176–1189. 10.1007/s00259-013-2652-z 24425423

[B28] Drzezga A. Barthel H. Minoshima S. Sabri O. (2014). Potential clinical applications of PET/MR imaging in neurodegenerative diseases. J. Nucl. Med. 55(Suppl. 2), 47S–55S. 10.2967/jnumed.113.129254 24819417

[B29] Dubois B. Feldman H. H. Jacova C. Hampel H. Molinuevo J. L. Blennow K.. (2014). Advancing research diagnostic criteria for Alzheimer's disease: the IWG-2 criteria. Lancet Neurol. 13, 614–629. 10.1016/S1474-4422(14)70090-0 24849862

[B30] Dukart J. Mueller K. Barthel H. Villringer A. Sabri O. Schroeter M. L. (2013). Meta-analysis based SVM classification enables accurate detection of Alzheimer's disease across different clinical centers using FDG-PET and MRI. Psychiatry Res. 212, 230–236. 10.1016/j.pscychresns.2012.04.007 23149027

[B31] Dukart J. Mueller K. Horstmann A. Barthel H. Möller H. E. Villringer A.. (2011). Combined evaluation of FDG-PET and MRI improves detection and differentiation of dementia. PLoS ONE 6:e18111. 10.1371/journal.pone.0018111 21448435 PMC3063183

[B32] Falini A. Bozzali M. Magnani G. Pero G. Gambini A. Benedetti B.. (2005). A whole brain MR spectroscopy study from patients with Alzheimer's disease and mild cognitive impairment. Neuroimage 26, 1159–1163. 10.1016/j.neuroimage.2005.03.005 15878675

[B33] Fan Y. Resnick S. M. Shen D. Kraut M. A. Davatzikos C. (2007). O2-03-06. Alzheimers Dement. 3(3 Suppl.), S190–S191. 10.1016/j.jalz.2007.04.031

[B34] Faraji-Dana Z. Tam F. Chen J. J. Graham S. J. (2016). A robust method for suppressing motion-induced coil sensitivity variations during prospective correction of head motion in fMRI. Magn. Reson. Imaging 34, 1206–1219. 10.1016/j.mri.2016.06.005 27451407

[B35] Fei B. Oleinick N. Wilson D. L. (2004). Registration of micro-PET and high-resolution MR images of mice for monitoring photodynamic therapy. SPIE 30, 371–379. 10.1117/12.535465

[B36] Frisoni G. B. Fox N. C. Jack C. R. Jr. Scheltens P. Thompson P. M. (2010). The clinical use of structural MRI in alzheimer disease. Nat. Rev. Neurol. 6, 67–77. 10.1038/nrneurol.2009.215 20139996 PMC2938772

[B37] Galvin J. Price J. Yan Z. Morris J. C. Sheline Y. I. (2011). Resting bold fMRI differentiates dementia with Lewy bodies vs. Alzheimer's disease. Neurology 76, 1797–1803. 10.1212/WNL.0b013e31821ccc83 21525427 PMC3100121

[B38] Goubran M. Douglas D. Chao S. Quon A. Tripathi P. Holley D.. (2015). Assessment of PET & ASL metabolism in the hippocampal subfields of MCI and AD using simultaneous PET-MR. EJNMMI Phys. 2(Suppl. 1):A73. 10.1186/2197-7364-2-S1-A73 26956334 PMC4798610

[B39] Hamelin L. Lagarde J. Dorothée G. Leroy C. Labit M. Comley R. A.. (2016). Early and protective microglial activation in Alzheimer's disease: a prospective study using ^18^F-DPA-714 PET imaging. Brain 139, 1252–1264. 10.1093/brain/aww017 26984188

[B40] Harada R. Okamura N. Furumoto S. Furukawa K. Ishiki A. Tomita N. (2015). ^18^F-THK5351: a novel PET radiotracer for imaging neurofibrillary pathology in Alzheimer's Disease. J. Nucl. Med. 57, 208–214. 10.1007/s00259-015-3035-4 26541774

[B41] Henriksen O. M. Marner L. Law I. (2016). Clinical PET/MR imaging in dementia and neuro-oncology. PET Clin. 11, 441–452. 10.1016/j.cpet.2016.05.003 27593248

[B42] Hui E. S. Fieremans E. Jensen J. H. Tabesh A. Feng W. Bonilha L.. (2012). Stroke assessment with diffusional kurtosis imaging. Stroke 43, 2968–2973. 10.1161/STROKEAHA.112.657742 22933581 PMC3479373

[B43] Jack C. R. Knopman D. S. Jagust W. J. Shaw L. M. Aisen P. S. Weiner M. W.. (2010). Hypothetical model of dynamic biomarkers of the Alzheimer's pathological cascade. Lancet Neurol. 9, 119–128. 10.1016/S1474-4422(09)70299-6 20083042 PMC2819840

[B44] Jadvar H. Colletti P. M. (2014). Competitive advantage of PET/MRI. Eur. J. Radiol. 83, 84–94. 10.1016/j.ejrad.2013.05.028 23791129 PMC3800216

[B45] Johnson K. A. Minoshima S. Bohnen N. I. Donohoe K. J. Foster N. L. Herscovitch P.. (2013). Appropriate use criteria for amyloid PET: a report of the amyloid imaging task force, the society of nuclear medicine and molecular imaging, and the Alzheimer's Association. J. Nucl. Med. 54, 476–490. 10.2967/jnumed.113.120618 23359661

[B46] Judenhofer M. S. Catana C. Swann B. K. Siegel S. B. Jung W. I. Nutt R. E. (2007). Simultaneous PET/MR images, acquired with a compact MRI compatible PET detector in a 7 Tesla magnet. Radiology 244, 807–814. 10.1148/radiol.2443061756 17709830

[B47] Karch C. M. Goate A. M. (2015). Alzheimer's disease risk genes and mechanisms of disease pathogenesis. Biol. Psychiatry 77, 43–51. 10.1016/j.biopsych.2014.05.006 24951455 PMC4234692

[B48] Krug R. Carballido-Gamio J. Banerjee S. Stahl R. Carvajal L. Xu D.. (2007). *In vivo* bone and cartilage MRI using fullybalanced steady-state free-precession at 7 tesla. Magn. Reson. Med. 58, 1294–1298. 10.1002/mrm.21429 17957777

[B49] Lewczuk P. Matzen A. Blennow K. Parnetti L. Molinuevo J. L. Eusebi P. M.. (2017). Cerebrospinal Fluid Aβ42/40 corresponds better than Aβ42 to amyloid PET in Alzheimer's disease. J. Alzheimers Dis. 55, 813–822. 10.3233/JAD-160722 27792012 PMC5147502

[B50] Li X. Li T. Q. Andreasen N. Wiberg M. K. Westman E. Wahlund L. O. (2014). The association between biomarkers in cerebrospinal fluid and structural changes in the brain in patients with Alzheimer's disease. J. Intern. Med. 275, 418–427. 10.1111/joim.12164 24237038

[B51] Lin W. Huang F. Duensing G. R. Reykowski A. (2012). High temporal resolution retrospective motion correction with radial parallel imaging. Magn. Reson. Med. 67, 1097–1105. 10.1002/mrm.23092 21842499

[B52] Mosconi L. Tsui W. H. Herholz K. Pupi A. Drzezga A. Lucignani G.. (2008). Multicenter standardized ^18^F-FDG PET diagnosis of mild cognitive impairment, Alzheimer's disease, and other dementias. J. Nucl. Med. 49, 390–398. 10.2967/jnumed.107.045385 18287270 PMC3703818

[B53] Navalpakkam B. K. Braun H. Kuwert T. Quick H. H. (2013). Magnetic resonance-based attenuation correction for PET/MR hybrid imaging using continuous valued attenuation maps. Invest. Radiol. 48, 323. 10.1097/RLI.0b013e318283292f 23442772

[B54] Nyberg S. Jönhagen M. E. Cselényi Z. Halldin C. Julin P. Olsson H. (2009). Detection of amyloid in Alzheimer's disease with positron emission tomography using [11C]AZD2184. Eur. J. Nucl. Med. Mol. Imaging 36, 1859–1863. 10.1007/s00259-009-1182-1 19495746 PMC2764092

[B55] Pan M. L. Mukherjee M. T. Patel H. H. Patel B. Constantinescu C. C. Mirbolooki M. R. (2016). Evaluation of [(11) C]TAZA for amyloid β plaque imaging in post-mortem human Alzheimer's disease brain region and whole body distribution in rodent PET/CT. Synapse 70, 163–176. 10.1002/syn.21893 26806100 PMC5358321

[B56] Pereznievas B. G. Stein T. D. Tai H. C. Dols-Icardo O. Scotton T. C. Barroeta-Espar I. (2013). Dissecting phenotypic traits linked to human resilience to Alzheimer's pathology. Brain 136, 2510–2526. 10.1093/brain/awt171 23824488 PMC3722351

[B57] Pichler B. J. Wehrl H. F. Kolb A. Judenhofer M. S. (2008). Positron emission tomography/magnetic resonance imaging: the next generation of multimodality imaging? Semin. Nucl. Med. 38, 199–208. 10.1053/j.semnuclmed.2008.02.001 18396179 PMC2762705

[B58] Querfurth H. W. Laferla F. M. (2010). Alzheimer's disease. N. Engl. J. Med. 362, 329–344. 10.1056/NEJMra0909142 20107219

[B59] Rabinovici G. D. Rosen H. J. Alkalay A. Kornak J. Furst A. J. Agarwal N.. (2011). Amyloid vs FDG-PET in the differential diagnosis of AD and FTLD. Neurology 77, 2034–2042. 10.1212/WNL.0b013e31823b9c5e 22131541 PMC3236517

[B60] Reiman E. M. Langbaum J. B. Fleisher A. S. Caselli R. J. Chen K. Ayutyanont N.. (2011). Alzheimer's prevention initiative: a plan to accelerate the evaluation of presymptomatic treatments. J. Alzheimers Dis. 26(Suppl. 3), 321–329. 10.3233/JAD-2011-0059 21971471 PMC3343739

[B61] Rezaei A. Defrise M. Nuyts J. (2014). ML-reconstruction for TOF-PET with simultaneous estimation of the attenuation factors. IEEE Trans. Med. Imaging 33, 1563–1572. 10.1109/TMI.2014.2318175 24760903

[B62] Rinne J. O. Brooks D. J. Rossor M. N. Fox N. C. Bullock R. Klunk W. E.. (2010). 11c-Pib PET assessment of change in fibrillar amyloid-beta load in patients with Alzheimer's disease treated with bapineuzumab: a phase 2, double-blind, placebo-controlled, ascending-dose study. Lancet Neurol. 9, 363–372. 10.1016/S1474-4422(10)70043-0 20189881

[B63] Rocchi L. Niccolini F. Politis M. (2015). Recent imaging advances in neurology. J. Neurol. 262, 2182–2194. 10.1007/s00415-015-7711-x 25808503

[B64] Rowe C. C. Ackerman U. Browne W. Mulligan R. Pike K. L. O'Keefe G.. (2008). Imaging of amyloid β in Alzheimer's disease with ^18^F-BAY94-9172, a novel pet tracer: proof of mechanism. Lancet Neurol. 7, 129–135. 10.1016/S1474-4422(08)70001-2 18191617

[B65] Rullmann M. Dukart J. Hoffmann K. T. Luthardt J. Tiepolt S. Patt M.. (2016). Partial-volume effect correction improves quantitative analysis of 18f-florbetaben β-amyloid pet scans. J. Nucl. Med. 57, 198–203. 10.2967/jnumed.115.161893 26541776

[B66] Sala-Llonch R. Bosch B. Arenaza-Urquijo E. M. Rami L. Bargalló N. Junqué C. (2011). Combining MRI modalities to study visual and default-mode networks in a-MCI. Adv. Alzheimers Dis. 2, 295–312. 10.3233/978-1-60750-793-2-295

[B67] Sander C. Y. Hooker J. M. Catana C. Normandin M. D. Alpert N. M. Knudsen G. M.. (2013). Neurovascular coupling to D2/D3 dopamine receptor occupancy using simultaneous PET/functional MRI. Proc. Nat. Acad. Sci. U.S.A. 110, 11169–11174. 10.1073/pnas.1220512110 23723346 PMC3703969

[B68] Schaefer A. Margulies D. S. Lohmann G. Gorgolewski K. J. Smallwood J. Kiebel S. J.. (2014). Dynamic network participation of functional connectivity hubs assessed by resting-state fMRI. Front. Hum. Neurosci. 8:195. 10.3389/fnhum.2014.00195 24860458 PMC4018560

[B69] Schlemmer H. P. Pichler B. J. Schmand M. Burbar Z. Michel C. Ladebeck R.. (2008). Simultaneous MR/PET imaging of the human brain: feasibility study. Radiology 248, 1028–1035. 10.1148/radiol.2483071927 18710991

[B70] Schuitemaker A. Kropholler M. A. Boellaard R. van der Flier W. M. Kloet R. W. van der Doef T. F. (2012). Microglial activation in Alzheimer's disease: an (R)-[11C]PK11195 positron emission tomography study - neurobiology of aging. Neurobiol. Aging 34, 128–136. 10.1016/j.neurobiolaging.2012.04.021 22840559

[B71] Seifert S. van der Lei G. van Dam H. T. Schaart D. R. (2013). First characterization of a digital sipm based time-of-flight PET detector with 1 mm spatial resolution. Phys. Med. Biol. 58, 3061–3074. 10.1088/0031-9155/58/9/3061 23587636

[B72] Shao Y. Cherry S. R. Farahani K. Meadors K. Siegel S. Silverman R. W.. (1997). Simultaneous PET and MR imaging. Phys. Med. Biol. 42, 1965–1970. 10.1088/0031-9155/42/10/010 9364592

[B73] Shcherbinin S. Schwarz A. J. Joshi A. D. Navitsky M. Flitter M. Shankle W. R. (2016). Kinetics of the Tau PET Tracer ^18^F-AV-1451 (T807) in subjects with normal cognitive function, mild cognitive impairment and Alzheimer's Disease. J. Nucl. Med. 57, 1535–1542. 10.2967/jnumed.115.170027 27151986

[B74] Shechter R. Schwartz M. (2013). Harnessing monocyte-derived macrophages to control central nervous system pathologies: no longer ‘if’ but ‘how’. J. Pathol. 229, 332–346. 10.1002/path.4106 23007711

[B75] Sun A. P. Kim J. H. Kim H. J. Kim T. E. Kim Y. J. Dong H. L. (2013). Preliminary study for a multicenter study of Alzheimer's disease cerebrospinal fluid biomarkers. Arch. Neur. 12, 1–8. 10.12779/dnd.2013.12.1.1

[B76] Tahmasian M. Pasquini L. Scherr M. Meng C. Förster S. Mulej Bratec S. (2015a). The lower hippocampus global connectivity, the higher its local metabolism in Alzheimer Disease. Neurology 84, 1956–1963. 10.1212/WNL.0000000000001575 25878180

[B77] Tahmasian M. Shao J. Meng C. Grimmer T. Diehlschmid J. Yousefi B. H.. (2015b). Based on the network degeneration hypothesis: separating individual patients with different neurodegenerative syndromes in a preliminary hybrid PET/MR study. J. Nucl. Med. 57, 410. 10.2967/jnumed.115.165464 26585059

[B78] Tang W. Kuruvilla S. A. Galitovskiy V. Pan M. L. Grando S. A. Mukherjee J. (2014). Targeting histone deacetylase in lung cancer for early diagnosis: ^18^F-FAHA PET/CT imaging of NNK-treated A/J mice model. Am. J. Nucl. Med. Mol. Imaging 4, 324–332. 24982818 PMC4074498

[B79] Tokuchi R. Hishikawa N. Sato K. Hatanaka N. Fukui Y. Takemoto M.. (2016). Differences between the behavioral and psychological symptoms of Alzheimer's disease and parkinson's disease. J. Neurol. Sci. 369, 278–282. 10.1016/j.jns.2016.08.053 27653908

[B80] Van der Wildt B. Wilhelmus M. M. Bijkerk J. Haveman L. Y. Kooijman E. J. Schuit R. C.. (2016). Development of carbon-11 labeled acryl amides for selective PET imaging of active tissue transglutaminase. Nucl. Med. Biol. 43, 232–242. 10.1016/j.nucmedbio.2016.01.003 27067043

[B81] Varrone A. Oikonen V. Forsberg A. Joutsa J. Takano A. Solin O. (2015). Positron emission tomography imaging of the 18-kda translocator protein (TSPO) with [18 Ffempa in Alzheimer's disease patients and control subjects. Eur. J. Nucl. Med. Mol. Imaging 42, 438–446. 10.1007/s00259-014-2955-8 25412766

[B82] Verhoeff N. P. Wilson A. A. Takeshita S. Trop L. Hussey D. Singh K. (2004). *In vivo* imaging of Alzheimer disease beta-amyloid with [11C]SB-13 PET. Am. J. Geriatr. Psychiatry 12, 584–595 10.1176/appi.ajgp.12.6.584 15545326

[B83] Visvikis D. Monnier F. Bert J. Hatt M. Fayad H. (2014). PET/MR attenuation correction: where have we come from and where are we going? Eur. J. Nucl. Med. Mol. Imaging 41, 1172–1175. 10.1007/s00259-014-2748-0 24633474

[B84] von Schulthess G. K. Veit-Haibach P. (2014). Workflow considerations in PET/MR imaging. J. Nucl. Med. 55(Suppl. 2):19S–24S. 10.2967/jnumed.113.129239 24790220

[B85] Werner P. Barthel H. Drzezga A. Sabri O. (2015). Current status and future role of brain PET/MRI in clinical and research settings. Eur. J. Nucl. Med. Mol. Imaging 42, 512–526. 10.1007/s00259-014-2970-9 25573629

[B86] Yanase D. Matsunari I. Yajima K. Chen W. Fujikawa A. Nishimura S.. (2005). Brain FDG PET study of normal aging in Japanese: effect of atrophy correction. Eur. J. Nucl. Med. Mol. Imaging 32, 794–805. 10.1007/s00259-005-1767-2 15759148

[B87] Yang Z. L. Zhang L. J. (2016). PET/MRI of central nervous system: current status and future perspective. Eur. Radiol. 26, 3534–3541. 10.1007/s00330-015-4202-5 26780640

[B88] Zhang K. Herzog H. Mauler J. Filss C. Okell T. W. Kops E. R.. (2014). Comparison of cerebral blood flow acquired by simultaneous [^15^O]water positron emission tomography and arterial spin labeling magnetic resonance imaging. J. Cereb. Blood Flow. Metab. 34, 1373–1380. 10.1038/jcbfm.2014.92 24849665 PMC4126098

[B89] Zhang Y. Wang S. Phillips P. Yang J. Yuan T. F. (2016). Three-dimensional eigenbrain for the detection of subjects and brain regions related with Alzheimer's disease. J. Alzheimers Dis. 50, 1163–1179. 10.3233/JAD-150988 26836190

[B90] Zheng L. J. Su Y. Y. Wang Y. F. Schoepf U. J. Varga-Szemes A. Pannell J.. (2017). Different hippocampus functional connectivity patterns in healthy young adults with mutations of APP/Presenilin-1/2 and APOEε4. Mol. Neurobiol. [Epub ahead of print]. 10.1007/s12035-017-0540-4 28502043

[B91] Zimmer E. Parent M. Leuzy A. Rowley J. Cheewakriengkrai L. Shin M. (2013). [18F]NAV4694 shows higher binding and wider dynamic range compared with [11C]Pib in Alzheimer's disease postmortem tissue. Alzheimers Dement. 9, 22–23. 10.1016/j.jalz.2013.05.026

[B92] Zsolt C. Maria Eriksdotter J. N. Anton F. Christer H. Per J. Magnus S. (2012). Clinical validation of ^18^F-AZD4694, an amyloid-β-specific PET radioligand. J. Nucl. Med. 53, 415–424. 10.2967/jnumed.111.094029 22323782

